# PlatypOUs—A Mobile Robot Platform and Demonstration Tool Supporting STEM Education

**DOI:** 10.3390/s22062284

**Published:** 2022-03-16

**Authors:** Melinda Rácz, Erick Noboa, Borsa Détár, Ádám Nemes, Péter Galambos, László Szűcs, Gergely Márton, György Eigner, Tamás Haidegger

**Affiliations:** 1 Research Centre for Natural Sciences, Eötvös Loránd Research Network, Magyar Tudósok krt. 2., H-1117 Budapest, Hungary; racz.melinda.9157@gmail.com (M.R.); gergely.marton@mindrove.com (G.M.); 2 János Szentágothai Doctoral School of Neurosciences, Semmelweis University, Üllői út 26, H-1085 Budapest, Hungary; 3 Selye János Doctoral College for Advanced Studies, Semmelweis University, Üllői út 22, H-1085 Budapest, Hungary; 4 Antal Bejczy Center for Intelligent Robotics, Robotics Special College, University Research and Innovation Center, Óbuda University, Bécsi út 96/B, H-1034 Budapest, Hungary; erick7327@gmail.com (E.N.); detar.borsa@gmail.com (B.D.); nemesgyadam@gmail.com (Á.N.); peter.galambos@irob.uni-obuda.hu (P.G.); laszlo.szucs@irob.uni-obuda.hu (L.S.); haidegger@irob.uni-obuda.hu (T.H.); 5 Biomatics and Applied Artificial Intelligence Institution, John von Neumann Faculty of Informatics, Óbuda University, Bécsi út 96/B, H-1034 Budapest, Hungary; 6 MindRove Kft., Hédervári út 43, H-9026 Győr, Hungary; 7 Faculty of Information Technology and Bionics, Pázmány Péter Catholic University, Práter utca 50/a, H-1083 Budapest, Hungary; 8 Physiological Controls Research Center, University Research and Innovation Center, Óbuda University, Bécsi út 96/B, H-1034 Budapest, Hungary

**Keywords:** STEM platform, custom mobile robot, electromyography-based control, Support Vector Machine Classification

## Abstract

Given the rising popularity of robotics, student-driven robot development projects are playing a key role in attracting more people towards engineering and science studies. This article presents the early development process of an open-source mobile robot platform—named PlatypOUs—which can be remotely controlled via an electromyography (EMG) appliance using the MindRove brain–computer interface (BCI) headset as a sensor for the purpose of signal acquisition. The gathered bio-signals are classified by a Support Vector Machine (SVM) whose results are translated into motion commands for the mobile platform. Along with the physical mobile robot platform, a virtual environment was implemented using Gazebo (an open-source 3D robotic simulator) inside the Robot Operating System (ROS) framework, which has the same capabilities as the real-world device. This can be used for development and test purposes. The main goal of the PlatypOUs project is to create a tool for STEM education and extracurricular activities, particularly laboratory practices and demonstrations. With the physical robot, the aim is to improve awareness of STEM outside and beyond the scope of regular education programmes. It implies several disciplines, including system design, control engineering, mobile robotics and machine learning with several application aspects in each. Using the PlatypOUs platform and the simulator provides students and self-learners with a firsthand exercise, and teaches them to deal with complex engineering problems in a professional, yet intriguing way.

## 1. Introduction

In science, technology, engineering and maths (STEM) education, particularly at advanced levels, laboratory demonstrations play a significant didactive role, as, on their account, students are able to inspect and test the available technologies in a specific domain. Our time’s megatrend, Robotics and Artificial Intelligence (AI), is inevitably changing the way we are acquiring, sharing and transferring knowledge [1,2,3]. Furthermore, robotics is believed to efficiently support our society regarding long-term sustainability [4]. We have seen great open science and research initiatives rising in various robotics domains from medical to underwater robotics and haptics [5,6,7,8], yet educational platforms are still not employed on a routine basis [9]. The recent speed at which robotic technologies have advanced (partially due to the coronavirus pandemic-induced global lockdowns) has also affected educational robotics [10].

Mobile robots deliver examples of the most comprehensive instruments for educators due to all the different engineering fields that are involved in achieving a variety of skill development tasks. This complexity level helps the STEM subjects to understand the relationship between the multiple disciplines, and how new technologies accumulated together increase the efficiency of systems, while reducing energy losses and improving materials and applied techniques. The literature provides tremendous examples where mobile robot platforms are used in educational settings, e.g., [11,12,13,14,15], where it can be appreciated how the education and training of students in the field of robotics is enhanced by giving them a hands-on experience on a physical platform. More concrete examples include the following:

- BSc level general courses, such as “Introduction to Robotics” at Óbuda University or the “Introduction to multi-agent and autonomous agent (robot) systems” at ELTE;
- MSc level “Robot Control Architectures” at BME and “Advanced Robot Control” or “Sensors and Actuators” at ÓU;
- National youth robot competitions, such as NJSZT’s (https://njszt.hu/hu/event/2022-01-23/magyar-ifjusagi-robot-kupa (accessed on 1 March 2022));
- Popular undergraduate robot competitions, such as Hungarians on the Mars (http://www.magyarokamarson.hu/weblap2019/index.php (accessed on 1 March 2022));
- World Robot Olympiad Junior category (https://wro.hu/robomission-kategoria/ (accessed on 1 March 2022)).

It is well recognized that there are already numerous similar platforms existing [16], yet it is important to emphasize based on the prior literature that there is significant value in creating educational robot platforms with local resources and custom attributes [17]. Similarities do exist with e.g., Sapientia University’s MARRtino project (https://www.marrtino.org/ (accessed on 1 March 2022)) [18] or the Industry 4.0 Master course at the University of Padova [19], yet the PlatypOUs is more focused on navigation and control, including bio-signal processing, fitting to the strategic research agenda of Óbuda University.

Regardless of the platforms already available on the market, it is useful for applied laboratories and STEM institutions to build their own prototypes because the development process can couple researcher and student communities. Furthermore, designing an open-source platform can channel and improve students’ knowledge and awareness in many subject areas. Today, complex engineering challenges can be resolved only by interdisciplinary teams, which is particularly applicable to the robotics domain [11]. It is also a third mission of our university to increase public outreach, and support the general understanding of modern technology, which is particularly important. Additional measures are required to popularize STEM, especially in our countries, where the demography shows a declining trend [20].

Capturing students’ interest in individual subjects is one of the major aims of this work. In this research, we introduce PlatypOUs, the innovative mobile robot platform designed at the Bejczy Antal Center of Intelligent Robotics of Óbuda University and the related Robotic Special College for educational purposes, which is supplied with modern sensors and the newest technology.

The goal of our project was twofold. On one hand, we developed a new mobile robot platform; furthermore, we created an eye-catching control system through which STEM involvement can be maximized. The robot successfully accomplished this purpose, since it was one of the main attractions of the 2021 European Researchers’ Night (https://ec.europa.eu/research/mariecurieactions/event/2021-european-researchers-night (accessed on 1 March 2022)) at our campus. More than 100 adolescent visitors (aged between 5 and 18) and their accompanying persons saw the robot in action; furthermore, a few of them tried the control system as well. In the next couple of months, our goal is to involve the robot in the STEM actions of the Robotics Special College of Óbuda University by reaching many high school students to persuade them to engage in the STEM fields.

The PlatypOUs project is one of the major projects of the Robotic Special College of Óbuda University. The college does have 30+ student members, from which ten students have been involved in the development of the robot and in exploring different applications of the platform, e.g., obstacle avoidance based on reinforcement learning and so on. Thus, the introduced solution is only one aspect where the robot is applied. Five students participated in the development of the platform itself (three BSc students—two males, one female (tasks: basic hardware, software, architecture, gazebo platform); one MSc student—male (rednode, Mindrove interfaces); one PhD student—male (ROS platform)). In the realization of the given EMG-based control system, another four students were involved (two MSc students—males (optimization of signal sending, realization of the communication within the components); three PhD students—two males, one gender-neutral person (SVM-based classifier, control application development), supervision of BSc, MSc students). The realization of the whole system as it is described below took approximately 7 months.

For the latter goal, we introduced a machine learning-based control solution that employed an available BCI device (MindRove) which captures EEG/EMG signals. While EEG headsets are extensively used as non-invasive interfaces in various functions from control to gaming, applying EEG signals for control purposes is not a minor task and requires comprehensive knowledge of both disciplines [21]. This work presents the first step made towards this goal, where we introduced the designed environments and an EMG-based controller, which was chosen for several reasons. In further research, we aimed to develop an interface that also utilizes the EEG signals. One of the largest challenges in achieving this goal was determining how the signals of the BCI device can be interpreted and applied. One potential solution is the machine learning-based signal classification, which is broadly used for feature detection in EMG and EEG signals [21], as well.

The control interface we designed is accessible and straightforward, so it is easy to use for every student during laboratory sessions. Unfortunately, regardless of the EEG–BCI type, there are a small number 15–30%) of users that cannot operate it for some reason, despite being healthy—this phenomenon is usually referred to as BCI illiteracy [22,23]. Consequently, we discarded the concept of a purely EEG–BCI. We chose to keep only the idea of utilizing an EEG measurement device for signal acquisition purposes, while the primary objective turned to the implementation of a much more easily applicable EMG-based system that uses signals that are most often regarded as artefacts—for instance, blinking or raising eyebrows.

## 2. Materials and Methods

### 2.1. Involved Technologies

We aimed to design an EMG-based controller with an AI-based interpreter that can translate signals to commands, understandable for our mobile robot platform. We used a MindRove BCI headset whose rich data content we were able to access through its SDK, which conforms to a common interface. This allowed the application of a Support Vector Machine (SVM)-based classifier. We considered various classifier possibilities; however, according to previous studies, SVMs can suffice in the case of small training databases [24], so we chose this solution. We designed a trainable SVM that can identify two different signals and apply them to render the user with two control commands based on the data collected via the MindRove headset. The first command allows switching directions, while the second command instructs the robot to move in the direction selected. The link between the headset and the robotic platform was created using the Message Queueing Telemetry Transport (MQTT) communication protocol [25]. MQTT is a lightweight protocol for IoT messaging and is widely used in robot control systems [26,27,28,29,30,31]. It was our choice because it has a small computational overhead and was easy to integrate into our system. To facilitate communication, we established a dedicated Local Area Network (LAN) and a MQTT broker (ISO/IEC 20922), which were necessary to handle the asynchronous information trade [32] between the EMG signals’ processing machine and the ROS node running in the computer of the robot, which gives velocity instructions to independently control each wheel. In such a case, Mosquitto MQTT broker is employed to maintain the scheduled communication between the machines involved [33].

### 2.2. The PlatypOUs Robot Platform

#### 2.2.1. Hardware

**Figure 1:**
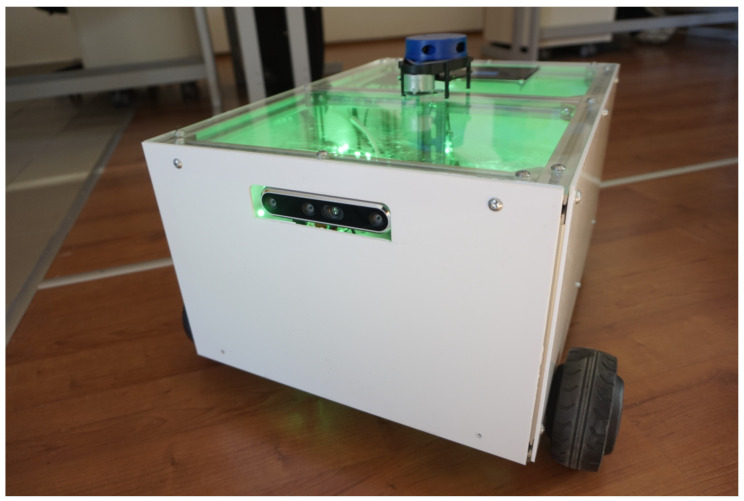
The PlatypOUs mobile robot in its first assembled format.

The PlatypOUs robot is a differential drive mobile platform (see Figure 1). It has two actively driven wheels, which are connected to brushless DC hub motors. The motors are controlled by an ODrive BLDC driver board, which is an open-source motor controlling solution, and the board schematic and the firmware used are freely available [34]. This board is linked to the main computer, Intel NUC8i5BEH mini PC, through a USB serial interface.

The robot’s power source is based on two 24 V, 15 Ah Li-ion battery packs, connected in parallel. The motor driver board is connected to the full battery voltage while the main computer and the sensors are powered by 12 V or 5 V, employing voltage regulators. All power wires have fuses, and the robot has a main switch as well as an emergency switch, which only disconnects the motor driver, so the robot cannot move but the computer stays turned on.

**Figure 2:**
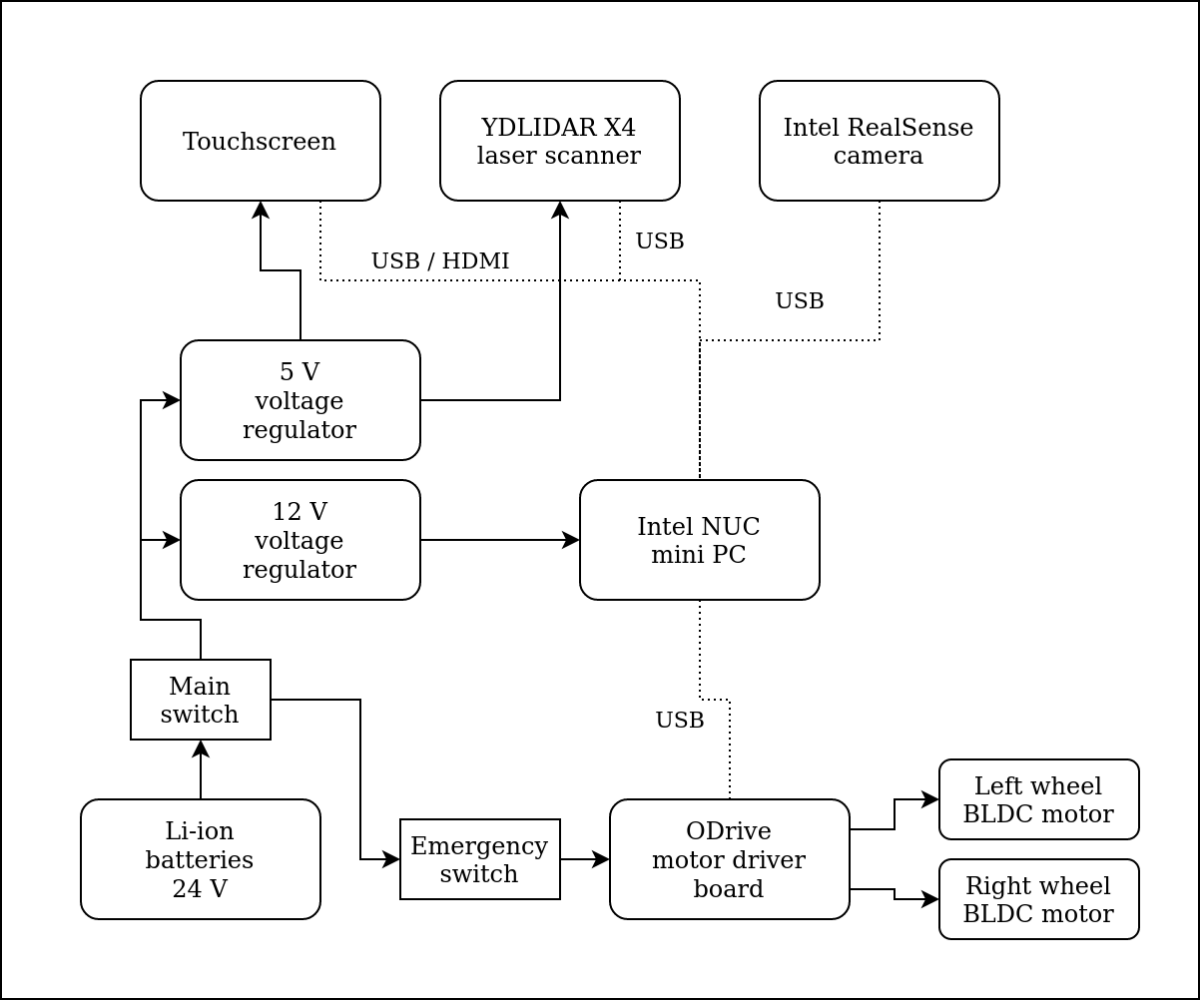
Hardware block diagram of the PlatypOUs mobile robot, including the core components and communication lines.

The robot has multiple sensors, a YDLIDAR X4 LIDAR (360-degree Laser Range Scanner with a maximum of 10 m range), an Intel RealSense D455 depth camera (with on-board IMU and depth error less than 2% at 4 m) and wheel encoders. The information from these sensors can be used to collect information about the environment of the robot, make a map of it, accurately determine the position of the robot, detect obstacles and automatically navigate to a desired point. For interfacing with the onboard computer, a touchscreen is attached to the top. A diagram of the hardware components and their connections can be seen in Figure 2.

The platform is made of aluminum rails in a rectangular shape with detachable plexiglass side panels. This is to make the design simple, easily extendable and modifiable. It also makes it easy to work on the hardware and the cabling. The top panel is transparent, so the inside is always visible, attracting the eyes of students.

#### 2.2.2. Software

The robot’s software is built using the open-source robotics middleware Robot Operating System (ROS) [35]. In an ROS system, the software components are called nodes and are running in independent processes, while the communication between them is made possible by the ROS core. The information is exchanged by messages sent to topics. A node can advertise a topic and publish messages on it, or subscribe to a topic and listen to messages.

To make the software environment portable and easily reproducible, the system uses Docker [36,37]. The software and all dependencies and setup steps are defined in the Dockerfile, which is used to generate an image. This image is running in a Docker container, separated from the host operating system.

In the system, every sensor, input, output and computation task has its own ROS node. From the wheel encoder measurements, odometry is calculated to estimate the robot’s position and velocity. With the laser scanner, the robot is capable of simultaneous localization and mapping (SLAM), using the gmapping ROS package (https://github.com/samialperen/oko_slam/blob/master/doc/gmapping_tutorial.md (accessed on 1 March 2022)). There is a web server running on the robot, which interacts with the ROS environment, and provides a web Graphical User Interface (GUI) that can be used to view the status of the robot along the generated map, to give the robot navigation goals or manually control it.

Motion control is achieved by a node that listens for messages containing velocity information. From the desired linear and angular velocities, it calculates the needed wheel speeds based on the wheel radius and the distance between the two wheels. It also communicates with the ODrive motor driver board on USB, and sends the velocities to it. The onboard firmware measures the wheel speeds and uses a PID control loop to reach the target value. It also sends the measured velocity values back to the node, which then publishes it back to the ROS system for use in odometry calculation.

#### 2.2.3. Programming and Simulation Environment

**Figure 3:**
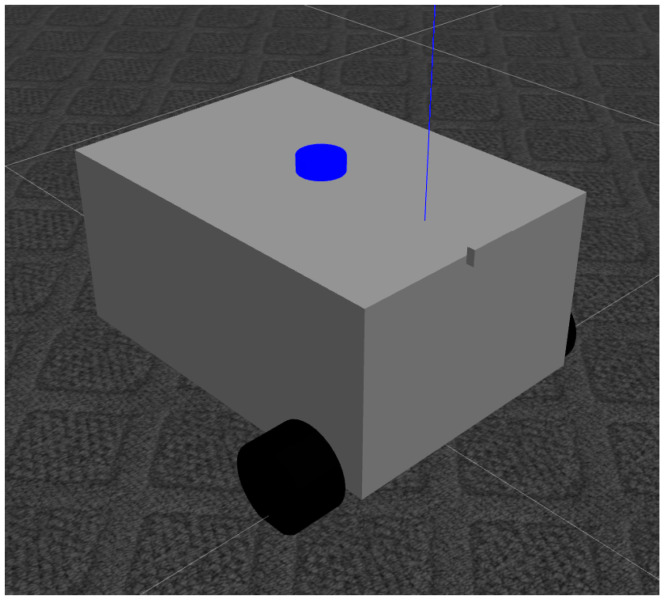
The model of the PlatypOUs mobile robot in the Gazebo simulator.

The PlatypOUs robot can be employed in the Gazebo simulator (http://gazebosim.org/ (accessed on 1 March 2022)) [38], in parallel. The simulation was designed to copy the actual robot as precisely as possible, in both the ROS interface and physical behavior. The simulated model has the same sensor sources as the real one, with very similar properties, and the same nodes can be run on it. This makes it possible to run nodes in either the simulation or the real robot, without modification. It is highly useful to be able to test components in the easy to use, fast and repeatable simulation, which then can be moved to the real-world hardware. Figure 3 shows the simulated robot in the Gazebo simulator.

### 2.3. Data Acquisition and Preprocessing

#### 2.3.1. Hardware

During laboratory practices, time is limited to the degree that completing even the mandatory tasks is not easy for students, who are usually lacking in experience. Therefore, we looked for a data acquisition device that is easy to install and use. Since EMG measurements do not require superior spatial resolution, we decided that a commercial EEG headset will suffice for our needs. Finally, the MindRove arc was chosen for our purposes. The device is designed to record the EEG signal of the brain; however, it is also able to record the EMG signal of face expressions—the latter have been used in this work.

**Figure 4:**
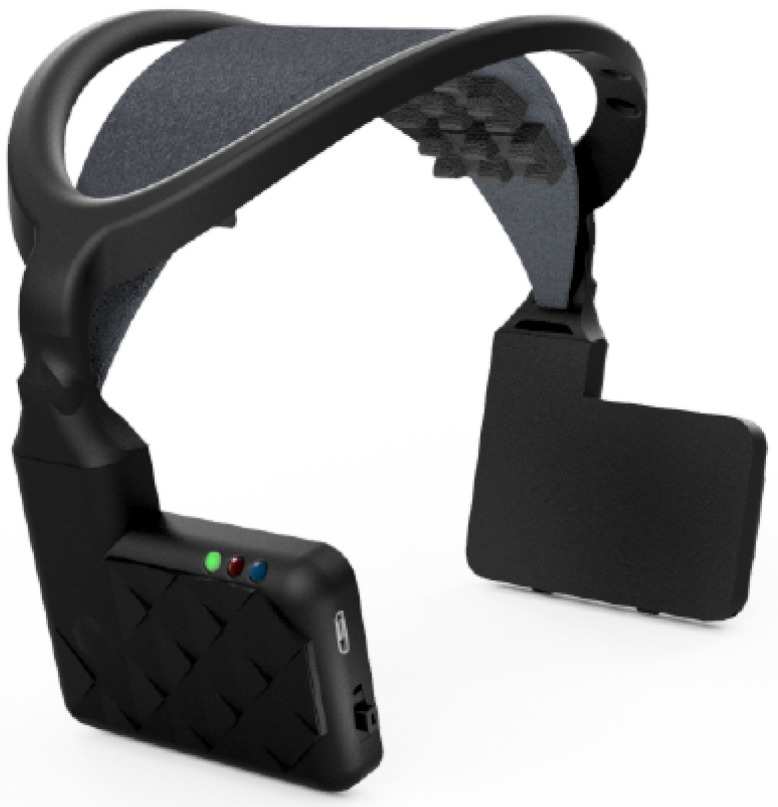
Picture of the MindRove. (Image credit: MindRove Inc.) arc [39].

The MindRove arc is a research-grade EEG headset developed by Hungarian startup MindRove (depicted in Figure 4). The device is intended for implementing event-related potential-based brain–computer interfaces, featuring semi-dry electrodes that enable fast setup and a Wi-Fi connection and ergonomic design that both enhance usability. The signal is acquired at 500 Hz in six channels, having 24-bit resolution [39]. If the device is worn as intended, electrode positions correspond to C5, C3, C1, C2, C4 and C6 in the international 10–20 system. The hardware is accompanied by a software development kit (SDK) [40] that is based on the BrainFlow framework [41] and supports building applications easily.

#### 2.3.2. Software Tools

**Figure 5:**
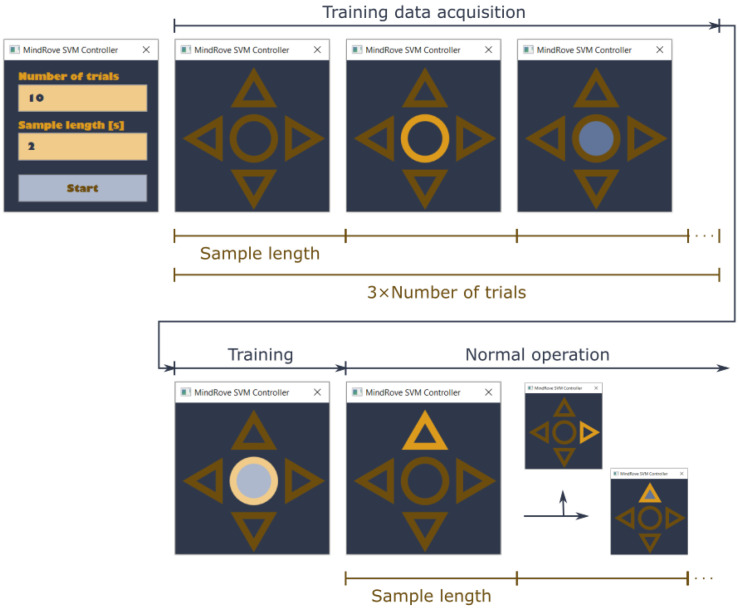
The operation of the app implementing data acquisition, classifier training and robot control (normal operation). Characteristic screenshots belonging to each phase are displayed: on the left side of the upper row, the start window can be seen with the default values of the input parameters (these settings are actually feasible for obtaining satisfactory results); in block “*Training data acquisition*”, possible cues are shown (for commands “*Stop*”, “*Switch direction*” and “*Go*”, respectively); on the left side of the bottom row, a separate sign indicates that training in progress; next to it, the initial state (“*Forward*”) is shown with two possible subsequent states (“*Turn right*” or “*Go forward*”).

The data acquisition and classifier training paradigm was implemented in C#, with the GUI written using Windows Presentation Foundation (WPF). Measurement data were accessed via the MindRove SDK. For complex computational tasks, we utilized Emgu CV (a C# wrapper for OpenCV, version 4.1.1) [42], both at the preprocessing (Fourier transform) and the classification (SVM training and evaluation) stages. A screenshot of the GUI is shown on the top left of Figure 5.

#### 2.3.3. Data Acquisition Paradigm

We implemented a simple mobile app that provides the training and operating environment for the classifier. Using the GUI, the user can specify the length of the control commands in seconds and the number of training instances per command. Considering the possible variations in electrode placement and the preferences/capabilities of the potential subjects, we decided that the classifier had to be trained each time the app starts. A great advantage of the SVM is that sufficient accuracy can be achieved using even 10–20 training samples per class. The optimal number of the training samples depends on the user and the chosen command (i.e., how accurately and uniformly they can repeat it, the degree of attention/focus during the training process, etc.); therefore, it can only be determined in an experimental, trial-and-error fashion.

After the user has provided the parameters necessary for training and clicked on the “*Start*” button, cues corresponding to the particular commands are presented. In the center of the screen, a dark blue circle with a brown border is displayed. This marks a “*Stop*” command, when the user remains idle. When its border turns to yellow, a “*Switch direction*” command is cued. When the inside of the circle turns to bright blue, a “*Go*” command has to be issued. Cues are in random order and are displayed during the whole trial (i.e., trials are not separated by, e.g., a blank screen).

When all data have been collected, both the border and the inside of the circle turn bright, signifying that training is in progress.

After the training phase, the circle turns dark again, showing that normal operation has started. The app follows the commands of the user and displays the result of the classification (i.e., from this point, no cue issued towards them). The active direction is marked by the only arrow with a yellow border and its inside turning bright blue indicates that the robot actually moves into that direction. The operation of the app is illustrated in Figure 5.

#### 2.3.4. Data Preprocessing

Control command classification is based on the power spectrum of the signal chunks (time windows) corresponding to the individual commands. Therefore, the classification is insensitive to the position (temporal displacement) of the EMG feature within a time window, but the presence of the entire feature is assumed in a particular sample. Thus, subsequent commands do not overlap in time.

Control commands take the shape of a $n_{s}\cdot f_{s}\times n_{ch}$ array, where $n_{s}$ is the sample/command length, $f_{s}=500$ Hz (the sampling frequency of the MindRove arc) and $n_{ch}=6$ (the number of EEG channels). Firstly, their discrete-time Fourier transform (DFT) is computed. Secondly, the power spectrum of the signal is taken. We used individual spectral components but only from the (0,80] Hz range. This domain comprehends frequency bands from delta to beta and includes most of the gamma band, which is sufficient for obtaining good classification performance while computation time (i.e., during training and evaluation) is kept relatively low. Finally, the standardization formula shown below is applied to normalize samples one-by-one using:

(1) $$x_{i}^{\prime }=\frac{x_{i}-\mu_{i}}{\sigma_{i}},$$

where $x_{i}^{\prime }$ and $x_{i}$ indicate the normalized and the original samples, while $\mu_{i}$ and $\sigma_{i}$ denote the average and the standard difference of the original sample, respectively. Standardization brings data variance to the same scale in each dimension, accelerating the training process.

#### 2.3.5. System Response

**Figure 6:**
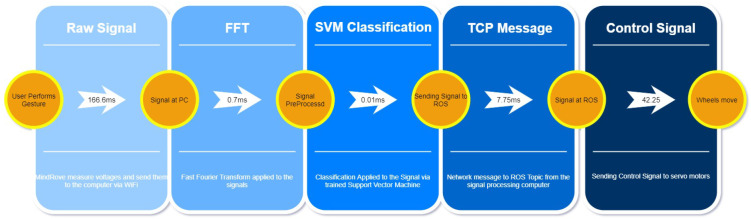
Time spans of sub-processes.

We measured the total response time of EMG control, by investigating each element from the command gesture to taking action by the end actuators. Visualization of the results can be seen in Figure 6. The architecture and components allow 217 ms control action velocity on an average basis.

#### 2.3.6. Signal Length

Instead of processing the EMG signals as continuous data, we defined the task as a simple classification problem, and separated the signal to a given length of parts. Then, we ran the SVM classification on the parts separately, one after the other. We investigated the effect of the length of the processed signal on the accuracy.

**Figure 7:**
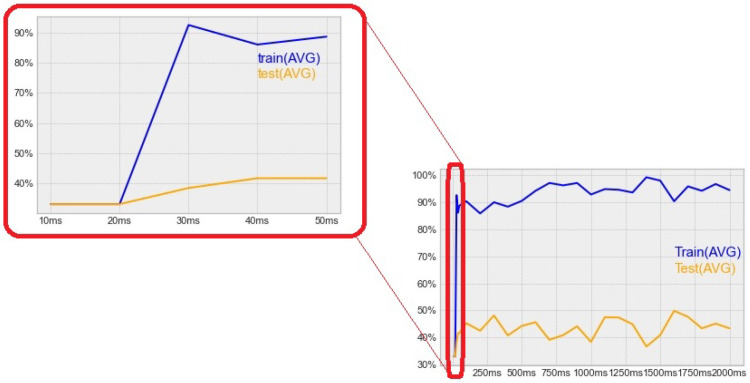
Classification accuracy by processed signal length. The right side shows the accuracy for different signal lengths from 10 ms to 2000 ms. On the left, the most critical part is highlighted.

The results of our measurements are visualized in Figure 7. We measured time spans from 10 ms to 2000 ms. The results showed that increasing the value over 30 ms does not improve the accuracy significantly. Thus, we decided to use the 30 ms length, which results in almost real-time processing.

### 2.4. SVM-Based Classifier

Support Vector Machine is a supervised classification technique where the clusters are separated using maximum-margin hyperplanes in the feature space. Points closest to these hyperplanes are termed support vectors [43]. We chose this method because it has a good generalization capability using small training databases. Given that each user has to train their own classifier and time during laboratory practices is a scarce resource, training databases cannot have an arbitrarily large size and training times are supposed to be short, as well. A further advantage of the particular implementation we used is that the type and parameter set of the algorithm are determined during the training. This feature can make the classifier more robust across various users and recording scenarios.

A possible drawback of SVM is that it is not able to extract spatial or frequency-related patterns, unlike, e.g., a convolutional neural network. It is questionable whether the benefits of the exploitation of these dependencies exceed the cost of using more data, but it is worth considering more complex machine learning methods in the future of this project.

### 2.5. Finalized EMG-Driven Architecture

The output of our classifier is an integer corresponding to the control command:

- 0—“*Stop*”
- 1—“*Switch direction*”
- 2—“*Go*”

**Figure 8:**
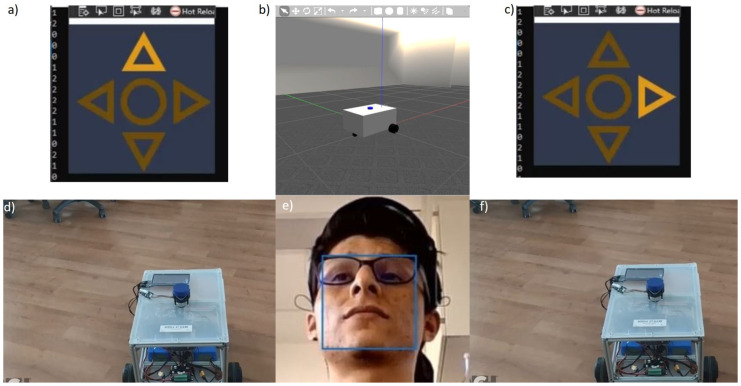
Previous (**a**,**d**) and succeeding (**c**,**f**) conditions of the real and simulated (**b**) PlatypOUs platform at the time when the user executes a change direction artefact by raising eyebrows (**e**).

**Figure 9:**
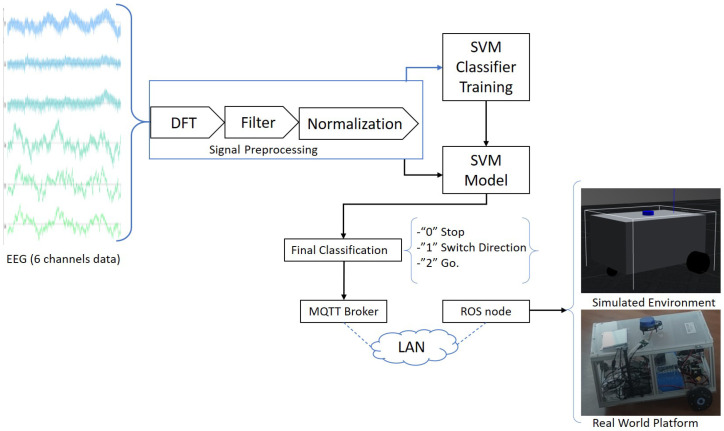
Flow of signal acquisition and categorization.

**Figure 10:**
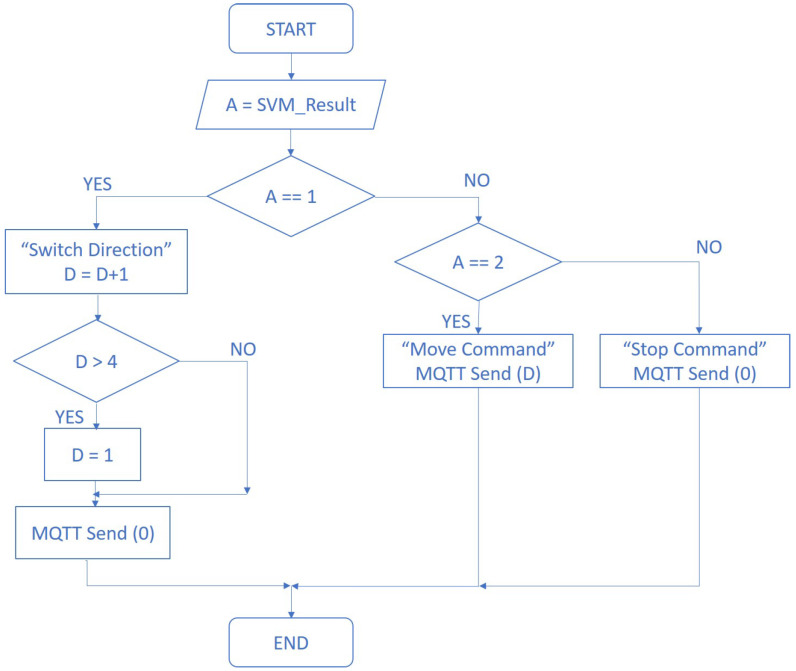
Flow diagram of the process of direction tracking.

Every time a “*Switch direction*” command arrives, e.g., as Figure 8 shows, the direction arrow in the interface takes a clockwise turn. The switch direction command is represented by a value of 1 coming as a result of the classification process. In order to make the system able to track this change, an extra variable was implemented to store the value that represents the direction that is currently selected in the application screen following the code shown below, as can be seen in the signal flow diagram in Figure 9 and the signal flow in Figure 10. The initial state of the system is the “*Forward*” direction (see Figure 8).

- 0—“*Forward*”
- 1—“*Right*”
- 2—“*Backwards*”
- 3—“*Left*”

The content of this variable is sent to the PlatypOUs platform through MQTT using a W-LAN, process that takes 7.75 ms, as can be seen referring to Figure 6. This method of communication was chosen due to the wide range of possibilities it brings to the project, as multiple teams are currently working on different aspects and applications for the mobile platform; MQTT allows us to standardise, in some way, the protocols that all the teams have at their disposal, especially with Internet of Things (IoT)-related projects.

Simultaneously, inside the mobile platform’s computer runs an ROS node that processes the information received from the classifier via the *MQTT “/test” topic*, and builds a “*twist” type message* with the wanted velocity values, which the node publishes in the ROS topic “*/cmd vel/eeg*” with values of 0.2 [m/s] for linear displacements and 0.5 [m/s] for rotational movements. The motor-driver node that activates the desired motion handles this message.

## 3. Procedure

In order to help the performance assessment of the classifier, our app saves the pre-trained SVM model into an XML file. For test purposes, we implemented a separate data acquisition app that is able to record numerous preprocessed training commands, and, along with their type, writes them into a CSV file. A routine that performs the evaluation of pre-recorded data using a pre-trained model was implemented, as well.

## 4. Results

### 4.1. SVM Classification and Assessment

Note that all the training sessions were performed by the same user during the development and test stages of this project. Considering that this work is in continuous development, an evaluation with a larger subject group is planned to take place in a later step. We organized our train–test sessions as follows. The subject was a 28-year-old male with vision corrected to normal. Applying one drop of saline solution on each electrode, we placed the headset above the parietal cortex; considering that the current results are our choices of control commands that were feasible to capture in this position and were easily discernible, as the “*Switch direction*” command, we used *eyebrow raising*, and as the “*Go*” command, *chewing* was applied (“*Stop*” was represented by rest). After training the classifier, using the same settings, we created a data table of 20 labeled samples per one command and then evaluated it using the pre-trained model.

**Figure 11:**
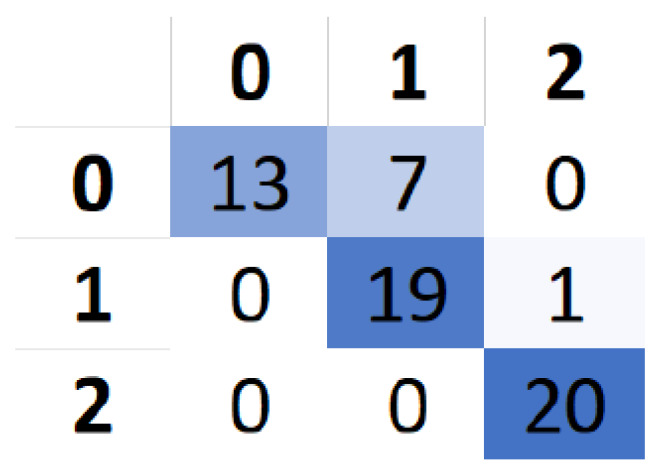
Confusion matrix, portraying the variety of commands predicted by the classifier. Rows correspond to the labels, columns to the predicted values. 0, 1, 2 indicate idle state, eyebrow raising and chewing, respectively.

Figure 11 demonstrates a confusion matrix, calculated by using results from a session executed by an experienced user. An accuracy of 86.67% could be achieved by selecting the control command setup described earlier.

### 4.2. Robot Control Tests—Real/Virtual

We tested the completed system inside the BARK laboratory environment and acquired the results presented below. The classification process is demonstrated in Figure 8, where the “*Change direction*” action is executed. In the center of the bottom row in Figure 8, it is evident that, in this case, the “*raising eyebrows*” command has been selected. From this figure, it is obvious that the platform stays in the unchanged position, while the chosen direction has altered by making a clockwise step, because the “*Go*” command has not been applied yet.

**Figure 12:**
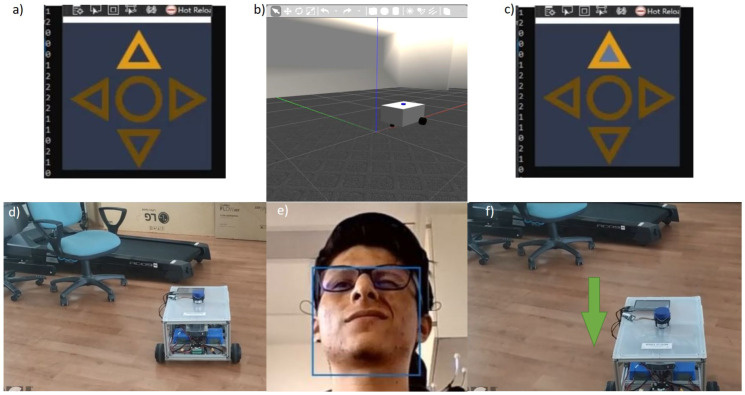
Previous (**a**,**d**) and succeeding (**c**,**f**) conditions of the real and simulated (**b**) PlatypOUs platform making a linear movement when the user executes a move artefact by winking (**e**).

**Figure 13:**
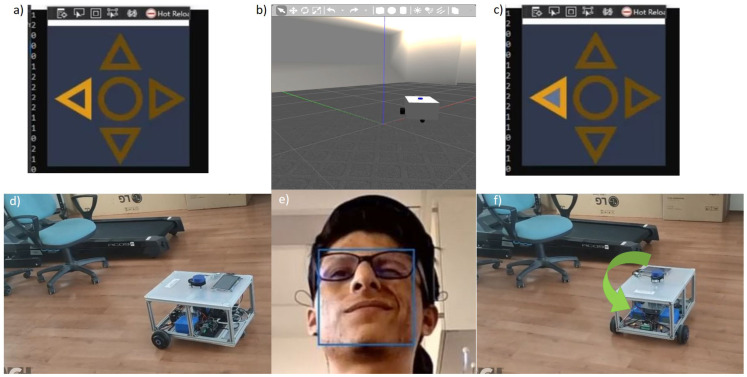
Previous (**a**,**d**) and succeeding (**c**,**f**) condition of the real and simulated (**b**) PlatypOUs platform making a rotational movement at the time when the user executes a move artefact by winking (**e**).

In order to observe the classification outcomes of the “*Go*” command in Figure 12 and Figure 13, a “*strong wink*” movement has been employed. In these figures, we can see that the PlatypOUs platform is performing *forward* and *rotation* actions, respectively.

The outcome of the classification procedure and the actions performed by the mobile platform in the real and simulated Gazebo environment can be observed in Figure 8, Figure 9, Figure 10, Figure 11, Figure 12 and Figure 13. As seen in the top center region of each figure (section b), the movements executed by the robot were identical in the simulation and the real world.

**Figure 14:**
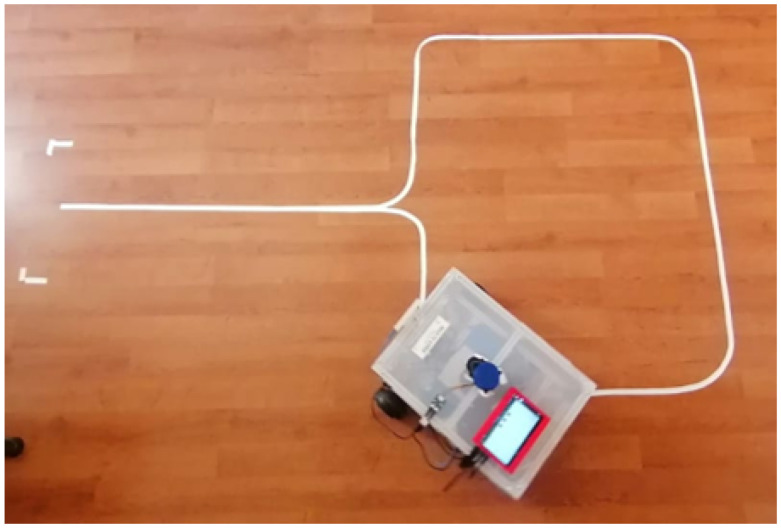
Shows the path created to test the behavior of the classifier and the platform response at the time of a driven session.

**Figure 15:**
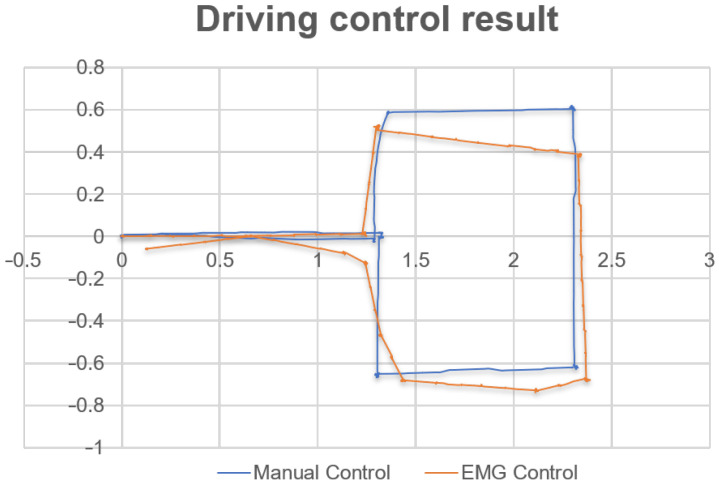
Comparison between a manual (in blue) and EMG (in orange) driving process.

In order to assess the performance of the final system, we defined a path the user would follow during evaluation tests. This particular sequence that involves both commands, to select the planned direction and to realize the motion, was planned in order to observe the behavior of the classifier together with the reaction of the mobile platform. The chosen pattern of movements can be recognized in Figure 14, where the system produced an average error of 12.39%, which matches the model evaluation in the previous part of this article. These results are further represented in Figure 15. While executing these tests, an important characteristic was observed regarding the response time of the system. From the moment the user performed a specific artefact command until the time the platform made the movement, we noticed an average time interval equal to 1.12(s), which can be assigned to the current data acquisition time that is previously set in the interface before the training process.

The usability of PlatypOUs was evaluated as an educational tool by seven students that were, or are currently, working on a project using the platform. The implementation of the NASA-TLX *Task Load Index* [44] allowed us to obtain feedback of how these students perceived the workload of the tasks they had while working on the platform.

**Figure 16:**
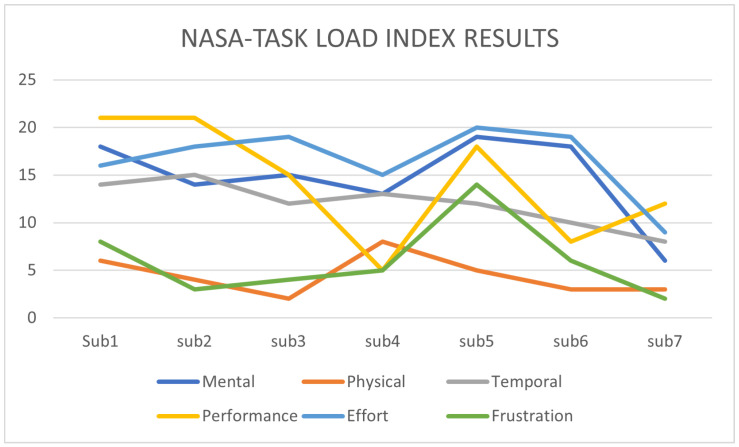
Results of the NASA-TLX assessment targeting the objective assessment of usability. Aggregated results of 7 subjects.

In Figure 16, it is notable how the temporal demand (in gray) tends to be moderately low, as well as the frustration level (in green) that the students passed through while they worked on the platform.

## 5. Discussion—Limitations and Future Work

During the project, it was experimentally shown that a platform for STEM-based education purposes can be successfully developed by a team of students with different backgrounds and various knowledge and abilities. The PlatypOUs platform will help student communities to attain a handful of skills in the future by allowing them to practice the implementation aspects of engineering theory.

The most challenging part of the project was the processing of bio-signals. SVM is capable of classifying such a signal, but its accuracy reduces with complexity. The improvements of deep learning give us more promising tools. For example, convolutional neural networks have proven to be useful in image processing, and in the past few years, they have also achieved promising results in processing brain signals [45]. Therefore, it would be a logical next step to change the SVM to a more complex, e.g., deep learning-based, architecture, as it may improve the accuracy of the signal processing significantly, possibly with only minimal additional time. During the assessment tests, we have determined that a cycle time of 0.1(s) is required to maintain the high-level control loop.

Besides changing the processing method, the other major improvement for the project would be—as mentioned before—to work with the EEG signals, preferably motor imagery signals to make the control more intuitive.

In the near future, extensive tests will be carried out with the system, both on the functional level and regarding usability and educational value as well. Sufficiently complex and appropriately designed user-centered validation tests are to be conducted, preferably within the frames of an open-house event, involving additional robot enthusiasts.

## 6. Conclusions

In our STEM-related applied robotics development project, PlatypOUs, the focus was placed on applied robotics and related skill acquisition. A physical platform was actually built, motion control enabled, and an SVM classifier was utilized to establish two various EMG-based face artefacts from MindRove BCI signals, moving eyebrows, blinking or chewing, in order to provide direction change and move commands to the PlatypOUs mobile platform.

The accuracy of the acquired classifier has been assessed via the application of 20 samples per command to the pre-trained model. The first observation made was that the accuracy of each model can vary drastically from one training process to another. This happens because of the variance of the contact points between the headset’s electrodes and the user’s head. It is also the reason that it was deemed necessary to train the classifier each time the paradigm runs. Regardless of the limitations, the user could acquire an accuracy of 86.667% in delivering their commends, which was good enough to execute steady control of the platform in a laboratory environment.

On the other hand, PlatypOUs has demonstrated the advantages of applying a mobile robot platform as an educational tool, giving students instant feedback on their work done on the platform, as other publications with similar objectives have already proven, e.g., [11,46,47], while encouraging them to participate in prototype contests in National Programs, e.g., [48,49,50]. It is imperative to mention that the platform we present in this paper, PlatypOUs, not only has the usual navigation stack that ROS has at its disposal but also a web server for control and supervision purposes, developed by one of the *design and construction* team members; moreover, the complete project has been encapsulated in a docker environment container to make it simpler for students to reproduce the entire system in their own computers by following a few steps that are mentioned in the GitHub of the project [51], giving them the capability to test their codes without wasting time in construction and long configuration processes, while we stick to our intention to create an open-source tool for laboratory practices.

The robot platform was designed using available open-source components for repeatability purposes. It incorporates an Intel RealSense D435i RGB-D camera and a YDLIDAR X4 LIDAR. The PlatypOUs was introduced in this article in detail, including its mechanical and functional components. The technologies that the platform is built on are diverse enough to demand knowledge from several subject fields. One of the greatest challenges was to acquire the data and preprocess the EMG signal.

PlatypOUs brings opportunities to STEM because of its custom and modular design. The price of the platform is approximately EUR 1200 (as of 2022 Q1). The mobile robot is currently equipped with basic navigation and mobility instrumentation, which can easily be amended in the future. In the next phase, we plan to extend the robot platform with new software features, powered by new sensors and mechanisms on both the physical and the simulation side. All the design and implementation was—and will be—executed by students, remaining to be a source of inspiration for the future generation of engineers.
